# Supplementary figures and images for: Metabolic and epigenetic dysfunctions underlie the arrest of in vitro fertilized human embryos in a senescent-like state
Source: PLoS Biol. 2022 Jun 30;20(6):e3001682. doi: 10.1371/journal.pbio.3001682 (PMC9246109; doi:10.1371/journal.pbio.3001682)

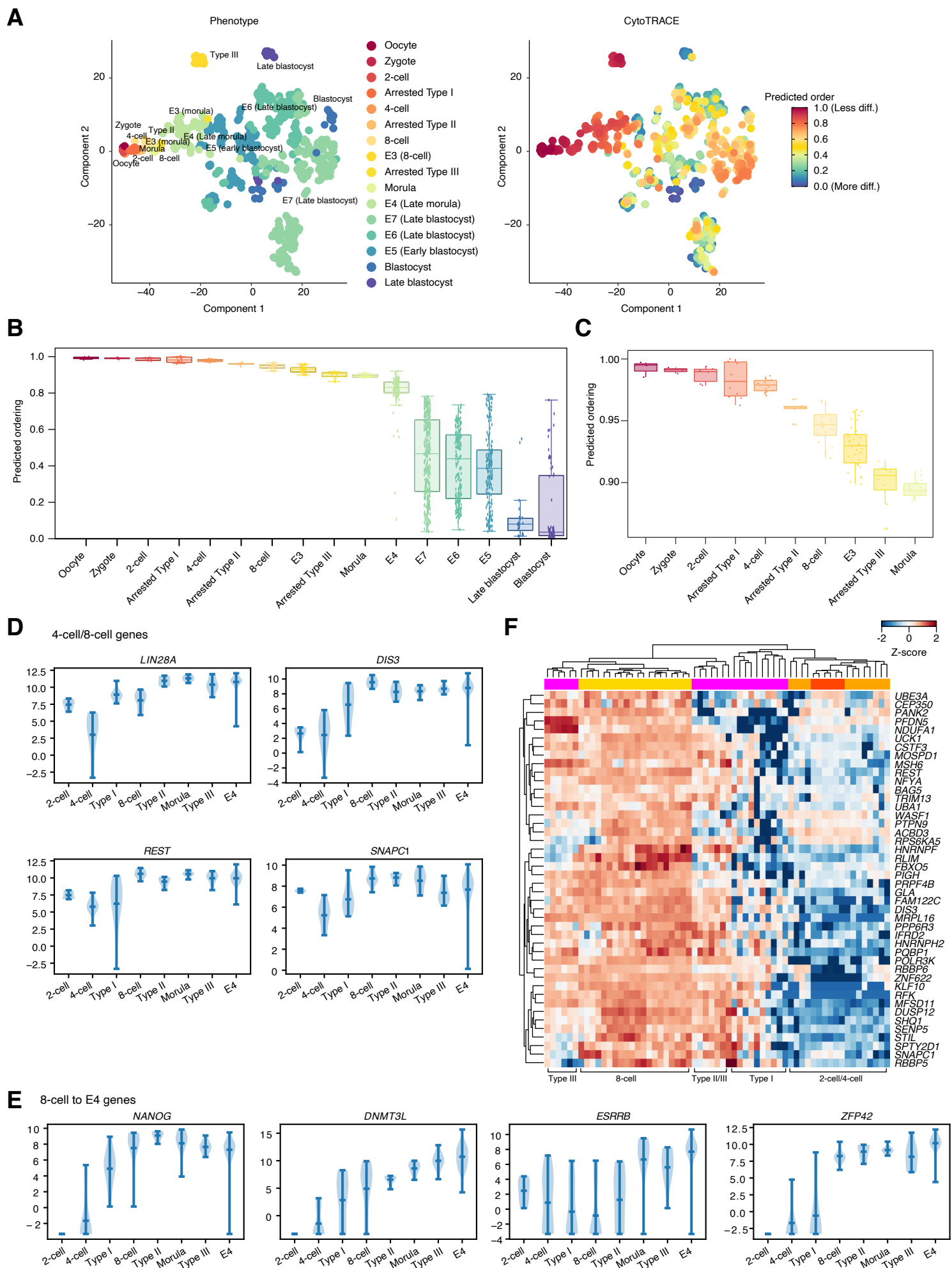

**S1 Fig**

Supplement: S1 Fig — Arrested embryos maintain developmental potential. (A) CytoTRACE cell embedding manifold, colored by cell type and embryo stage (left) or by predicted developmental order (right). A predicted developmental order score of 1.0 is less differentiated, and a score of 0.0 is more. E = Embryonic-stage samples, as defined in [19], for this and all subsequent figures. (B) Box plots of the predicted ordering from CytoTRACE for all cells/embryos at the indicated stages, ordered by the mean developmental predicted ordering. Each dot is a cell/embryo. (C) As in panel B, but only showing the stages from oocyte to morula. (D) Violin plots showing expression of 4-cell/8-cell-specific human genes. Underlying data can be found in: https://figshare.com/articles/dataset/Human_embryo_normalized_gene_expression_data/19775992. (E) Violin plots showing expression of 8-cell/E4-specific human genes, i.e., developmental genes involved in the establishment of the blastocyst. Underlying data can be found in: https://figshare.com/articles/dataset/Human_embryo_normalized_gene_expression_data/19775992. (F) Heatmap of the Z-scores of expressions of the genes in the 8-cell signature identified in [26]. Underlying data can be found in: https://figshare.com/articles/dataset/Human_embryo_normalized_gene_expression_data/19775992. All of the panels derived from CytoTRACE use non-normalized tag counts from: https://figshare.com/articles/dataset/Human_embryo_normalized_gene_expression_data/19775992. (PDF) [file pbio.3001682.s006.pdf]

**A**

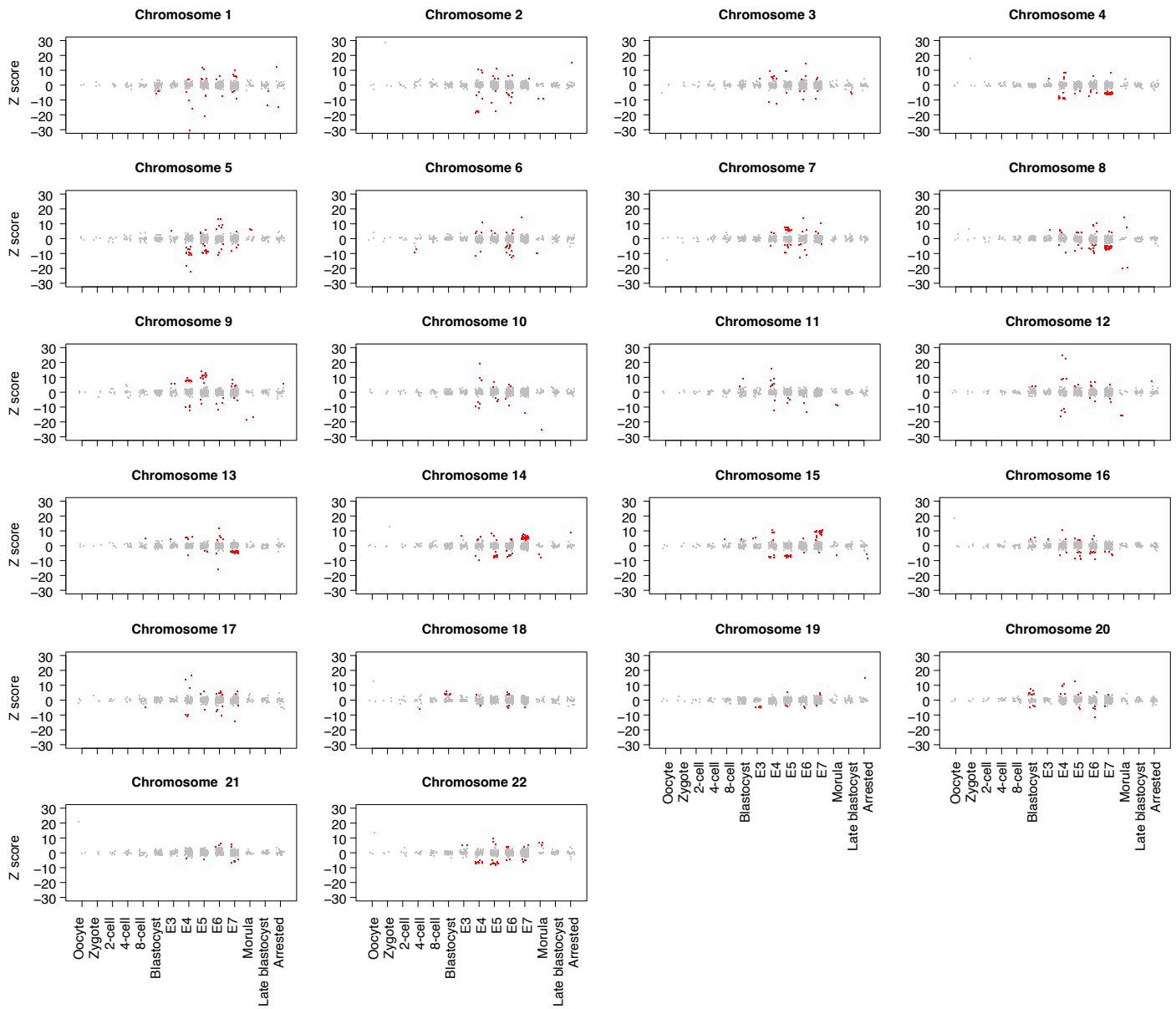

**B**

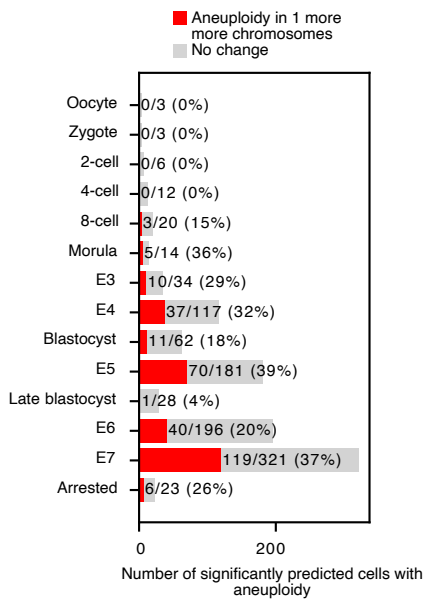

**C**

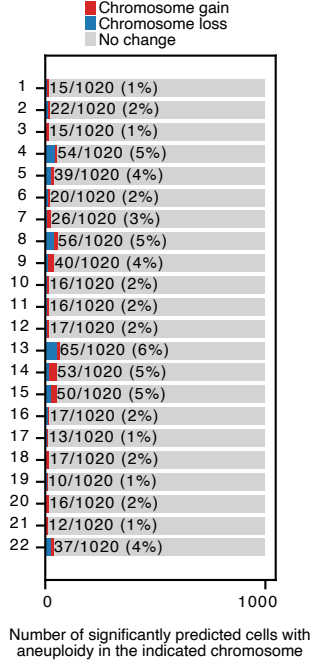

**D**

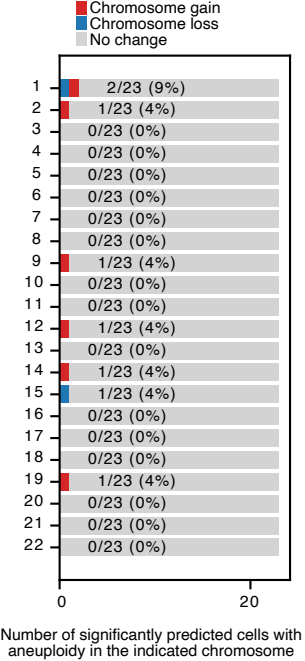

Supplement: S2 Fig — Arrested embryos are (mainly) karyotypically normal. (A) Karyotype abnormalities estimated using the approach outlined in [31]. In these plots, each chromosome is plotted separately, and the gray dots are each cell/embryo in the indicated stage of development or arrest. Red dots indicate when the gene expression on that chromosome exceeds the Z-score threshold and is significantly over or under represented. Red dots are indicative of aneuploidies, and those above the line suggest a gain of a chromosome or part of a chromosome, and those below the line suggest a loss of a chromosome or part. (B) Bar chart showing the predicted aneuploidies in the indicated developmental stages. Red bars indicate cells/embryos with a predicted loss or gain of a chromosome, while those in gray are predicted to be normal. (C) Percentage of the aneuploidies observed, broken down by chromosome in the normal embryo dataset. (D) As in panel C, but only the arrested embryos. All of these panels use data underlying results from S2 Data. (PDF) [file pbio.3001682.s007.pdf]

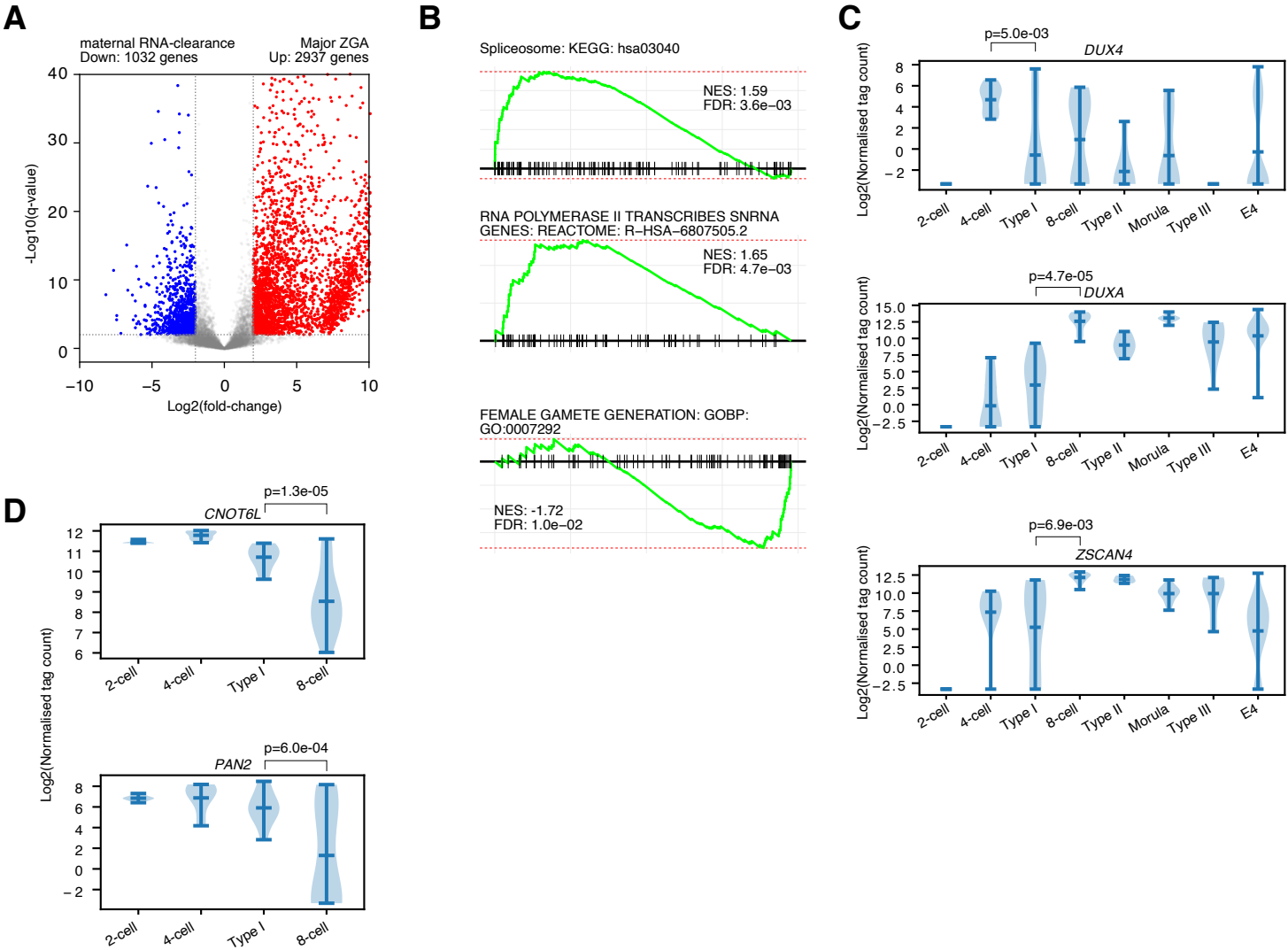

S3 Fig

Supplement: S3 Fig — Type I arrested embryos have MZT problems. (A) Volcano plot showing the fold-change versus significance when comparing 2-cell-stage embryos versus 8-cell-stage. Differential expression was calculated using DESeq2, and a minimum fold-change of 4, and a q-value of 0.01 was considered significantly different. The q-value is the Bonferroni–Hochberg multiple test corrected p-value. We defined “maternal RNA-clearance genes” as those that were significantly down-regulated, and “major ZGA genes” as those that were up-regulated. Significantly DE up-regulated genes are labeled in red and down-regulated genes in blue. The number of genes passing the differential expression thresholds are marked on the plot. DE genes/TEs are listed in S3 Data. (B) GSEA for the up- and down-regulated genes as ranked in panel A. Underlying data can be found in S3 Data. (C) Violin plots for the expression of key major ZGA genes, DUX4, DUXA, and ZSCAN4. Significance is from a 2-sided Welch’s t test. Underlying data can be found in: https://figshare.com/articles/dataset/Human_embryo_normalized_gene_expression_data/19775992. (D) Violin plots for the expression of critical maternal RNA clearance genes CNOT6L and PAN2. Significance is from a 2-sided Welch’s t test. Underlying data can be found in: https://figshare.com/articles/dataset/Human_embryo_normalized_gene_expression_data/19775992. DE, differentially expressed; GSEA, gene set enrichment analysis; MZT, maternal-to-zygotic transition; TE, transposable element; ZGA, zygotic genome activation. (PDF) [file pbio.3001682.s008.pdf]

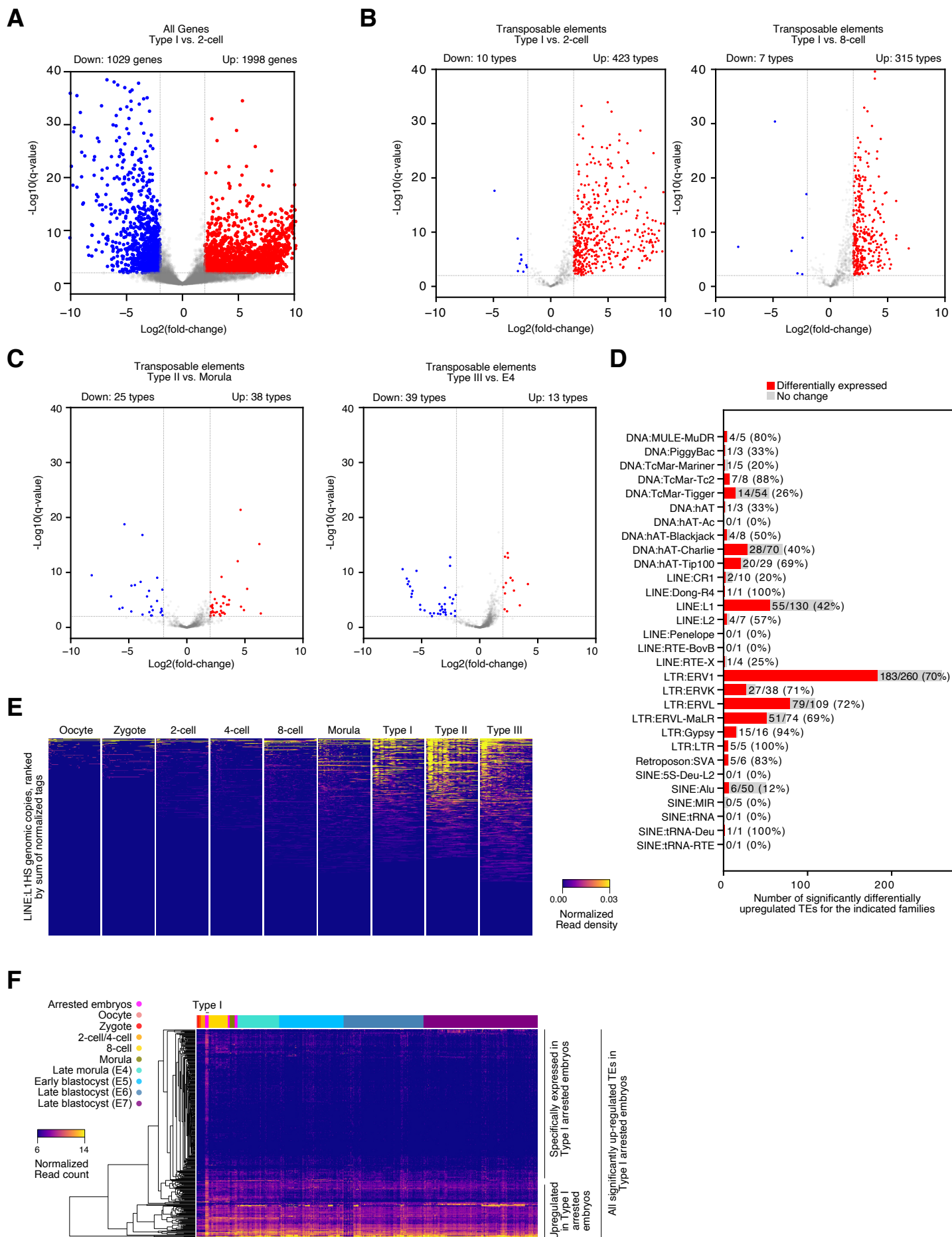

Supplement: S4 Fig — Transposable element expression is disturbed in Type I arrested embryos. (A) Volcano plot for all genes when comparing Type I arrested embryos to 2-cell-stage cells. Differential expression was calculated using DESeq2, and a minimum fold-change of 4, and a q-value of 0.01 was considered significantly different. The q-value is the Bonferroni–Hochberg multiple test corrected p-value. Significantly DE up-regulated genes are labeled in red and down-regulated genes in blue. The number of genes passing the differential expression thresholds are marked on the plot. DE genes are listed in S4 Data. (B) Volcano plot, as in panel A, but only containing TE types, and comparing Type I arrested embryos to 2-cell-stage embryos (left volcano), or 8-cell-stage embryos (right volcano). DE TEs are listed in S4 Data. (C) Volcano plot, as in panel A, but only containing TE types, and comparing Type II arrested embryos to morula-stage embryos (left volcano), or Type III arrested embryos versus E4 (early blastocyst)-stage embryos (right volcano). DE TEs are listed in S4 Data. (D) Number of differentially regulated TE types for the indicated TEs, when comparing Type I arrested embryos to 4-cell-stage embryos. DE TEs are listed in S4 Data. (E) Heatmaps of the RNA-seq read tag density for all genomic copies of LINE L1HS copies (rows). Heatmaps are the density of normalized tag counts (in reads per million) for each sample and are ranked by the sum of each row for each heatmap. The raw data for this plot is available from GSA under the accession HRA001406. The genome locations for the LINE L1HS are available for download from the UCSC genome browser. (F) Heatmap for the expression of all differentially regulated TEs in the Type I arrested embryos. The rows are the 315 TEs identified in panel B, the columns are the arrested embryos and all stages form Oocyte to late blastocyst. The location of the Type I embryos is indicated on the top row. DE TEs are listed in S4 Data. DE, differentially expr [file pbio.3001682.s009.pdf]

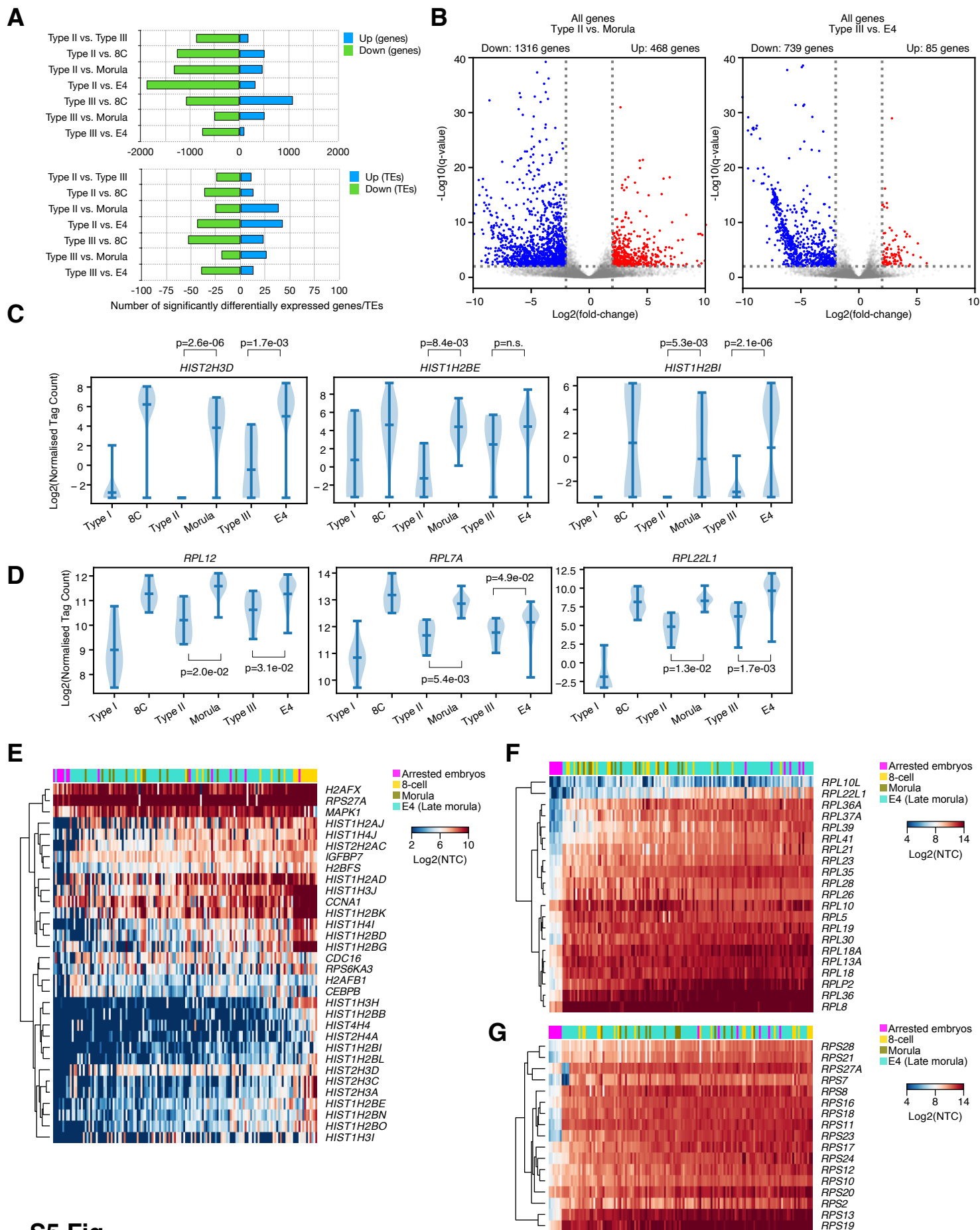

S5 Fig

Supplement: S5 Fig — Arrested embryos have reduced ribosome and nucleosome expression. (A) Number of significantly DE genes (top chart) and TEs (bottom chart) (a fold-change of at least 4, and a Bonferroni–Hochberg corrected q-value of less than 0.01) in the indicated comparisons. Underlying data can be found in S1 Data. (B) Volcano plots showing all genes when comparing Type II versus morula (left) or Type III versus E4 (right)-stage embryos. Differential expression was calculated using DESeq2, and a minimum fold-change of 4, and a q-value of 0.01 was considered significantly different. The q-value is the Bonferroni–Hochberg multiple test corrected p-value. Significantly DE up-regulated genes are labeled in red and down-regulated genes in blue. The number of genes passing the differential expression thresholds are marked on the plot. DE genes are listed in S4 Data. (C) Violin plot for the expression of selected histones. Significance is from a 2-sided Welch’s t test. Underlying data can be found in: https://figshare.com/articles/dataset/Human_embryo_normalized_gene_expression_data/19775992. n.s. = not significant. (D) Violin plot for selected large or small ribosome subunits. Significance is from a 2-sided Welch’s t test. Underlying data can be found in: https://figshare.com/articles/dataset/Human_embryo_normalized_gene_expression_data/19775992. (E) Heatmap of the expression of all histones/nucleosomes and selected senescence-related genes (from the REACTOME category: SENESCENCE−ASSOCIATED SECRETORY PHENOTYPE (SASP): REACTOME: R−HSA−2559582.2). Expression is presented as log2 NTC. Underlying data can be found in: https://figshare.com/articles/dataset/Human_embryo_normalized_gene_expression_data/19775992. Heatmap of all significantly DE (fold-change >4 and q-value <0.01) large ribosome subunits. Expression is presented as log2 NTC. Underlying data can be found in: https://figshare.com/articles/dataset/Human_embryo_normalized_gene_expression_data/19775992. (F) As in panel E, but for all [file pbio.3001682.s010.pdf]

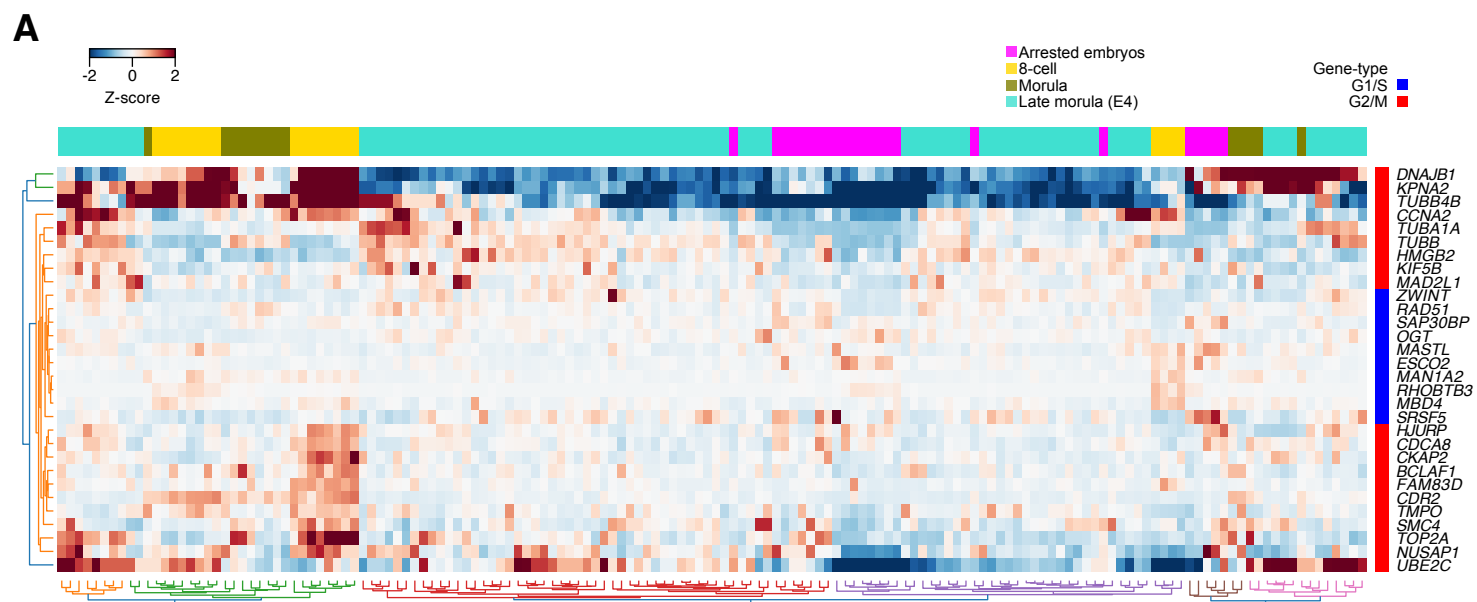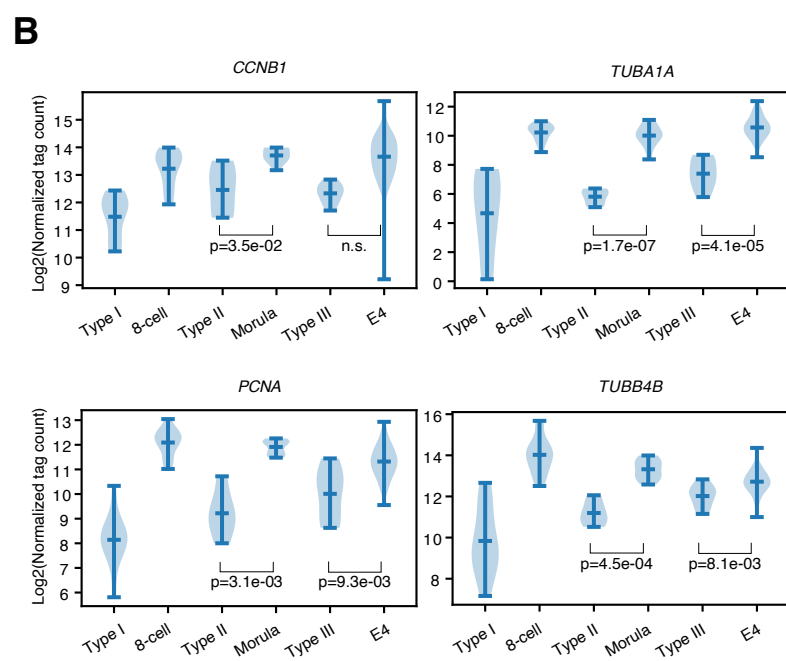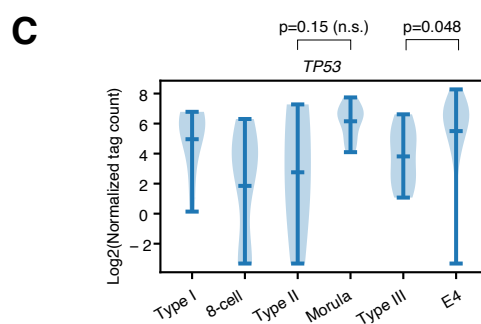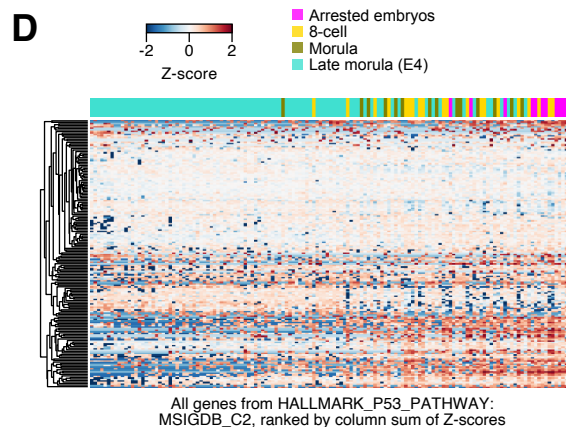

**S6 Fig**

Supplement: S6 Fig — Arrested embryos have decreased expression of cell cycle genes. (A) Heatmap showing the expression of selected cell cycle–related genes. The cell/embryo stage is indicated in the top, colored bar legend. Genes specifically expressed in G1/S or G2/M to a cell cycle phase (as defined in [48]) are marked on the right-hand side of the heatmap. Underlying data can be found in: https://figshare.com/articles/dataset/Human_embryo_normalized_gene_expression_data/19775992. (B) Violin plots showing expression of cell cycle–related gene, CCNB1, PCNA, and the tubulin subunits TUBA1A and TUBB4B. Significance is from a 2-sided Welch’s t test. Underlying data can be found in: https://figshare.com/articles/dataset/Human_embryo_normalized_gene_expression_data/19775992. (C) Violin plot for the expression of TP53 (p53). Significance is from a 2-sided Welch’s t test. Underlying data can be found in: https://figshare.com/articles/dataset/Human_embryo_normalized_gene_expression_data/19775992. n.s. = not significant. (D) Heatmap for the expression of p53 target genes (HALLMARK_P53_PATHWAY set), ranked by the sum of the columns. Each column is a single cell or embryo, and the arrested embryos are labeled in pink. Underlying data can be found in: https://figshare.com/articles/dataset/Human_embryo_normalized_gene_expression_data/19775992. (PDF) [file pbio.3001682.s011.pdf]

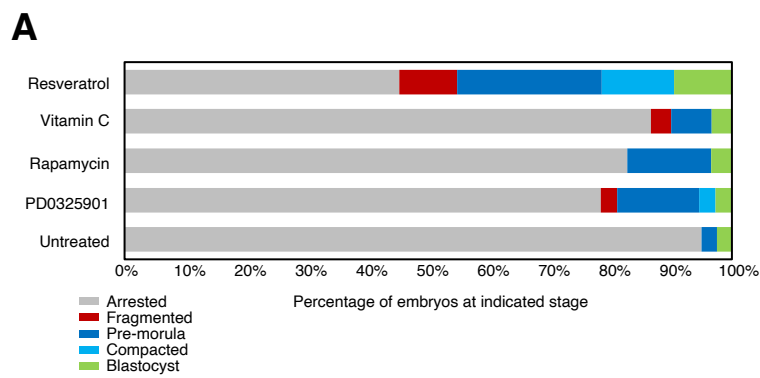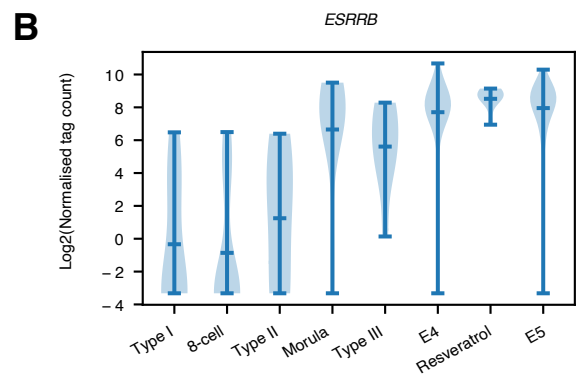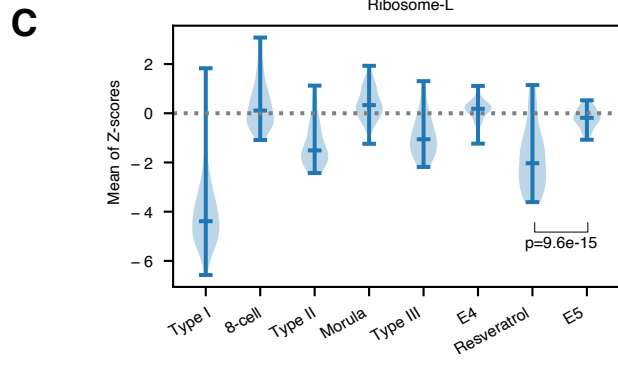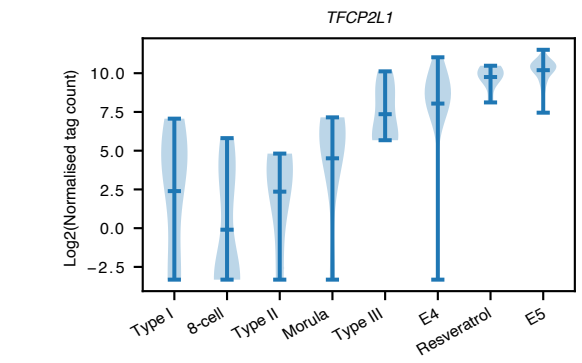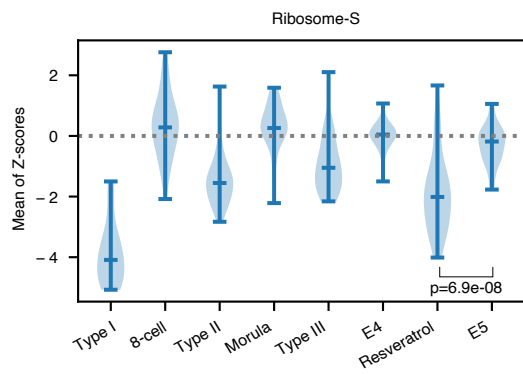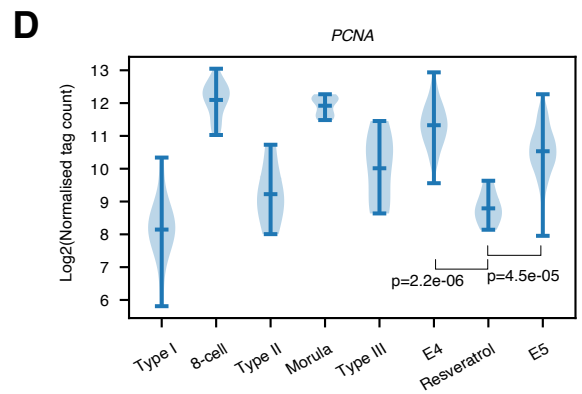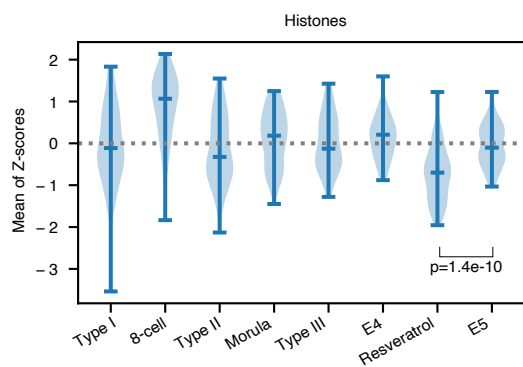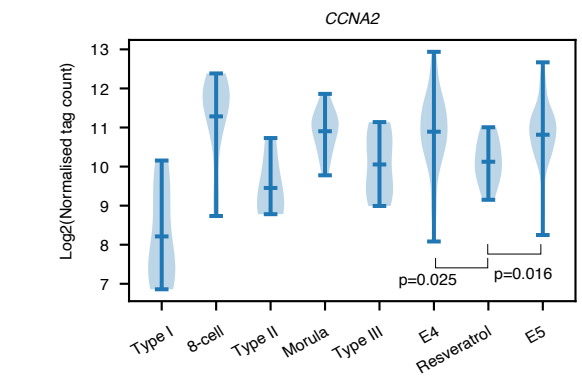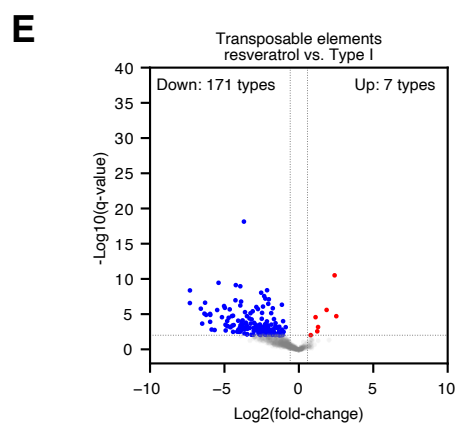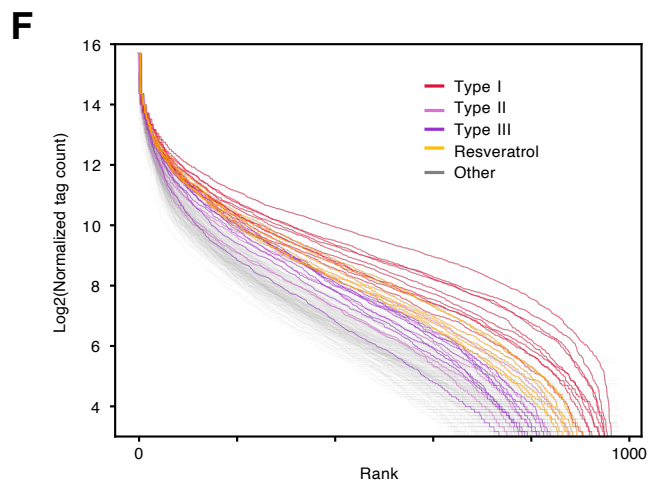

**S7 Fig**

Supplement: S7 Fig — Treatment of arrested embryos with small molecules, and resveratrol corrects developmental and cell cycle problems, but not ribosomes and nucleosome expression. (A) Percentage of arrested embryos that recommenced development, and the stage they reached, when treated with the indicated small molecules. Underlying data can be found in S1 Data. (B) Violin plot showing the expression of the blastocyst-related genes ESRRB and TFCP2L1 in the indicated embryonic stages and in arrested and resveratrol-treated embryos. Underlying data can be found in: https://figshare.com/articles/dataset/Human_embryo_normalized_gene_expression_data/19775992. (C) Violin plots showing the distribution of Z-scores of expression for all the small and large ribosomes and histone genes in the indicated embryonic stages and in arrested and resveratrol-treated embryos. Significance is from a 2-sided Welch’s t test. Underlying data can be found in: https://figshare.com/articles/dataset/Human_embryo_normalized_gene_expression_data/19775992. (D) Violin plots showing the expression of the cell cycle–related genes PCNA and CCNA2 in the indicated embryonic stages and in arrested and resveratrol-treated embryos. Significance is from a 2-sided Welch’s t test. Underlying data can be found in: https://figshare.com/articles/dataset/Human_embryo_normalized_gene_expression_data/19775992. (E) Volcano plot showing all significantly differentially expressed (fold-change >4 and q-value <0.01) for all genes and TEs when comparing resveratrol-treated versus Type I arrested embryos. Differential expression was calculated using DESeq2, and a minimum fold-change of 4, and a q-value of 0.01 was considered significantly different. The q-value is the Bonferroni–Hochberg multiple test corrected p-value. Significantly DE up-regulated genes are labeled in red and down-regulated genes in blue. The number of genes passing the differential expression thresholds are marked on the plot. Underlying data can be found in S4 Data. (F) [file pbio.3001682.s012.pdf]

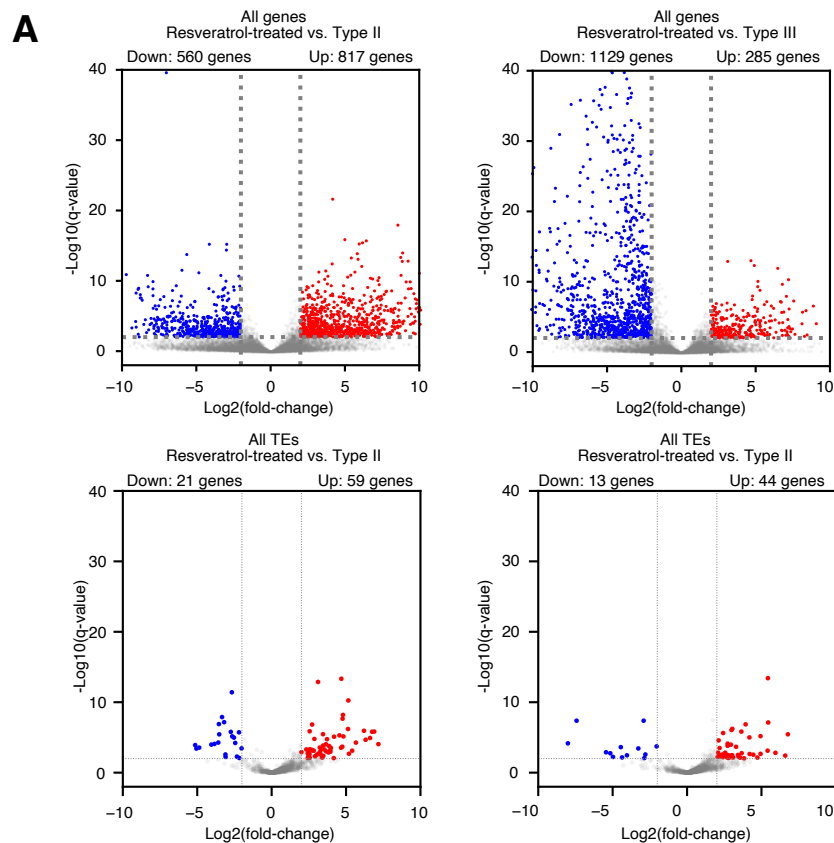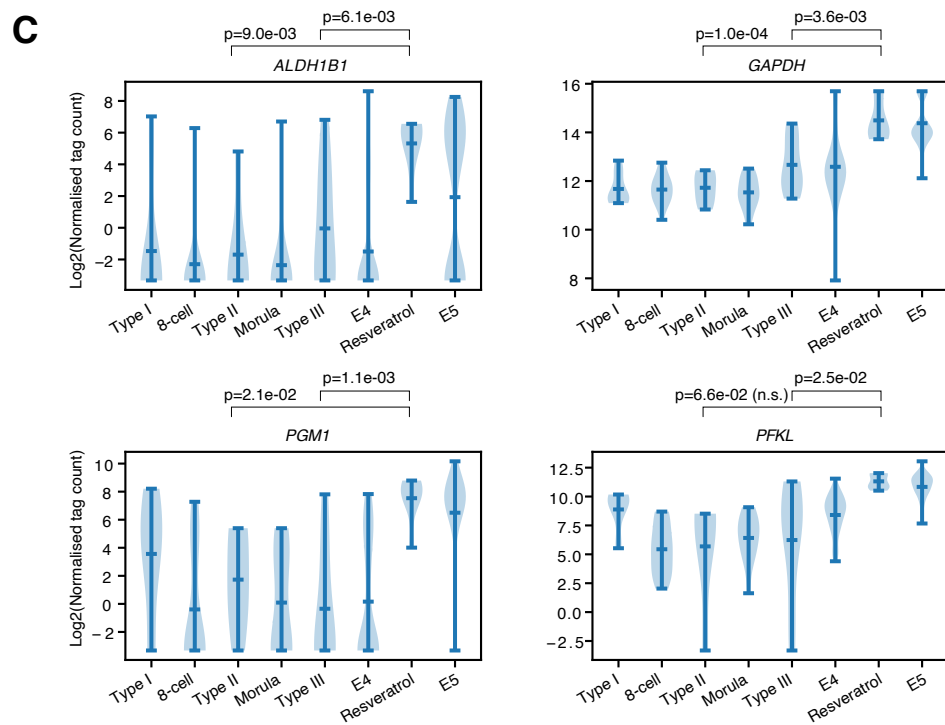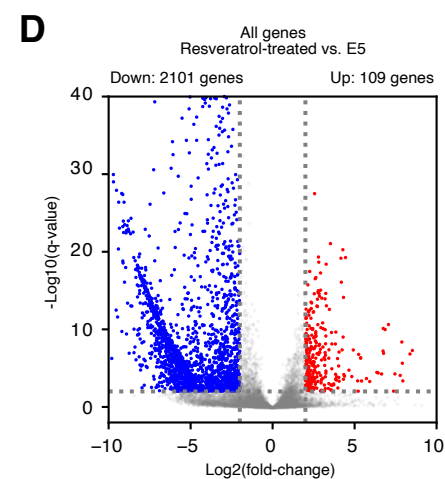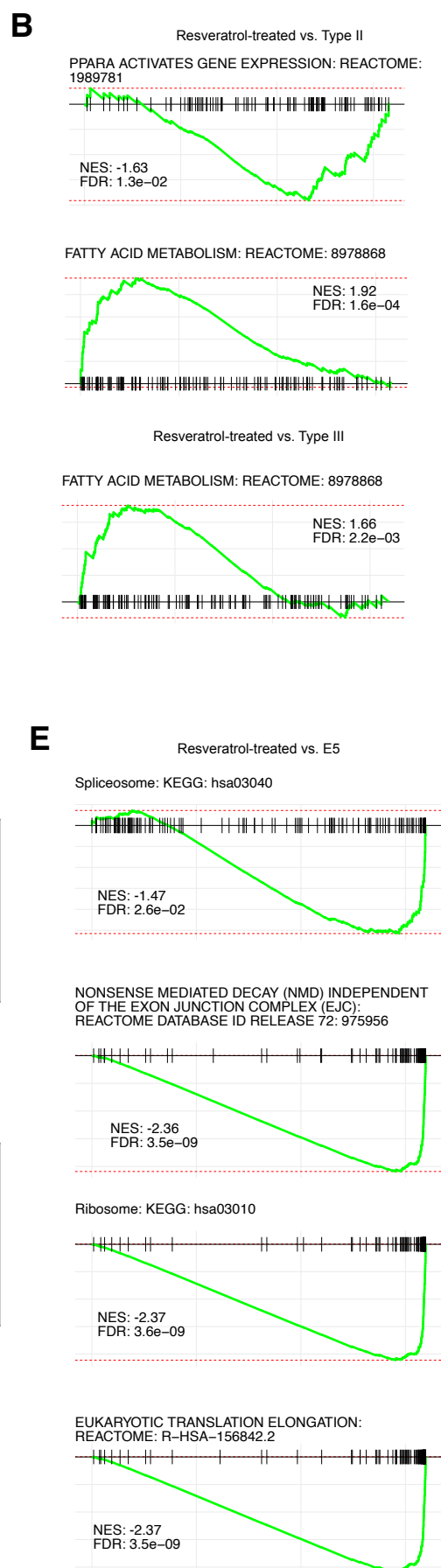

**S8 Fig**

Supplement: S8 Fig — Comparison of resveratrol-treated versus other embryonic cells. (A) Volcano plot showing all significantly differentially expressed (fold-change >4 and q-value <0.01) for all genes and TEs when comparing showing resveratrol versus Type II (left) or Type III (right) arrested embryos. Differential expression was calculated using DESeq2, and a minimum fold-change of 4, and a q-value of 0.01 was considered significantly different. The q-value is the Bonferroni–Hochberg multiple test corrected p-value. Significantly DE up-regulated genes are labeled in red and down-regulated genes in blue. The number of genes passing the differential expression thresholds are marked on the plot. DE genes/TEs are listed in S4 Data. (B) GSEA showing significantly different terms for resveratrol versus Type II arrested embryos. Underlying data can be found in S5 Data. (C) Violin plots showing the expression of the indicated glycolysis-related genes ALDH1B1, GAPDH, PGM1, and PFKL. Significance is from a 2-sided Welch’s t test. Underlying data can be found in: https://figshare.com/articles/dataset/Human_embryo_normalized_gene_expression_data/19775992. n.s. = not significant. (D) Volcano plots (as in panel A), but showing resveratrol-treated embryos versus E5-stage embryos. DE genes are listed in S4 Data. (E) GSEA of the ranked DE genes from panel D. Underlying data can be found in S5 Data. DE, differentially expressed; GSEA, gene set enrichment analysis; TE, transposable element. (PDF) [file pbio.3001682.s013.pdf]

**A**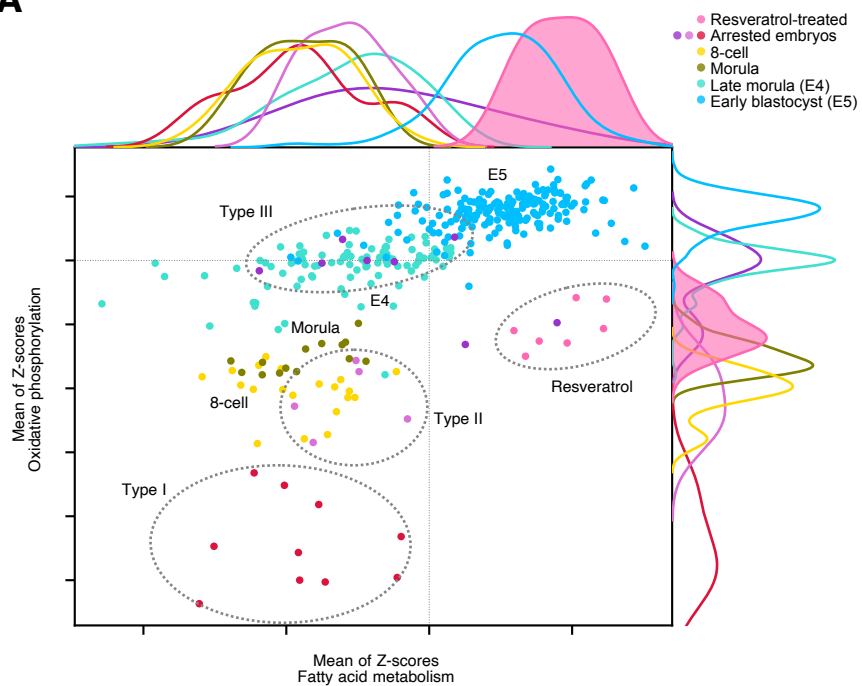**B**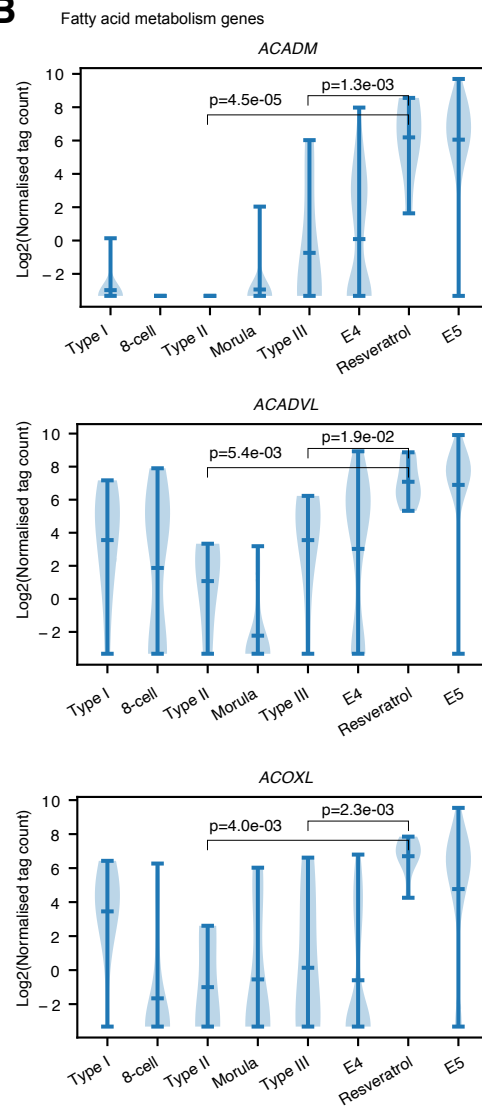**C**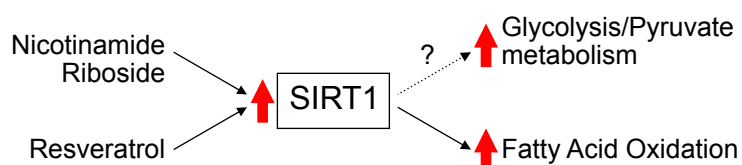**S9 Fig**

Supplement: S9 Fig — Resveratrol up-regulates the expression of fatty acid metabolic genes. (A) 2D dot plot showing the sum of the Z-scores for the genes in the indicated KEGG categories. The x-axis scores the fatty acid metabolism pathway, the y-axis the oxidative phosphorylation pathway. Each dot in the plot is a cell/embryo, and the top and right axis have the kernel density for each group of cells. The resveratrol-treated embryos have a filled in color (pink) for emphasis. The arrested and resveratrol-treated embryos are indicated by dashed lines, and the normal developmental states are indicated by labels. Underlying data can be found in S1 Data. (B) Violin plots showing the expression of selected fatty acid-related metabolic genes. Significance is from a 2-sided Welch’s t test. Underlying data can be found in: https://figshare.com/articles/dataset/Human_embryo_normalized_gene_expression_data/19775992. (C) A model for the action of resveratrol and NR on SIRTs and metabolic pathways. The dotted arrow between SIRT1 and glycolysis implies indirect regulation. NR, nicotinamide riboside. (PDF) [file pbio.3001682.s014.pdf]

A

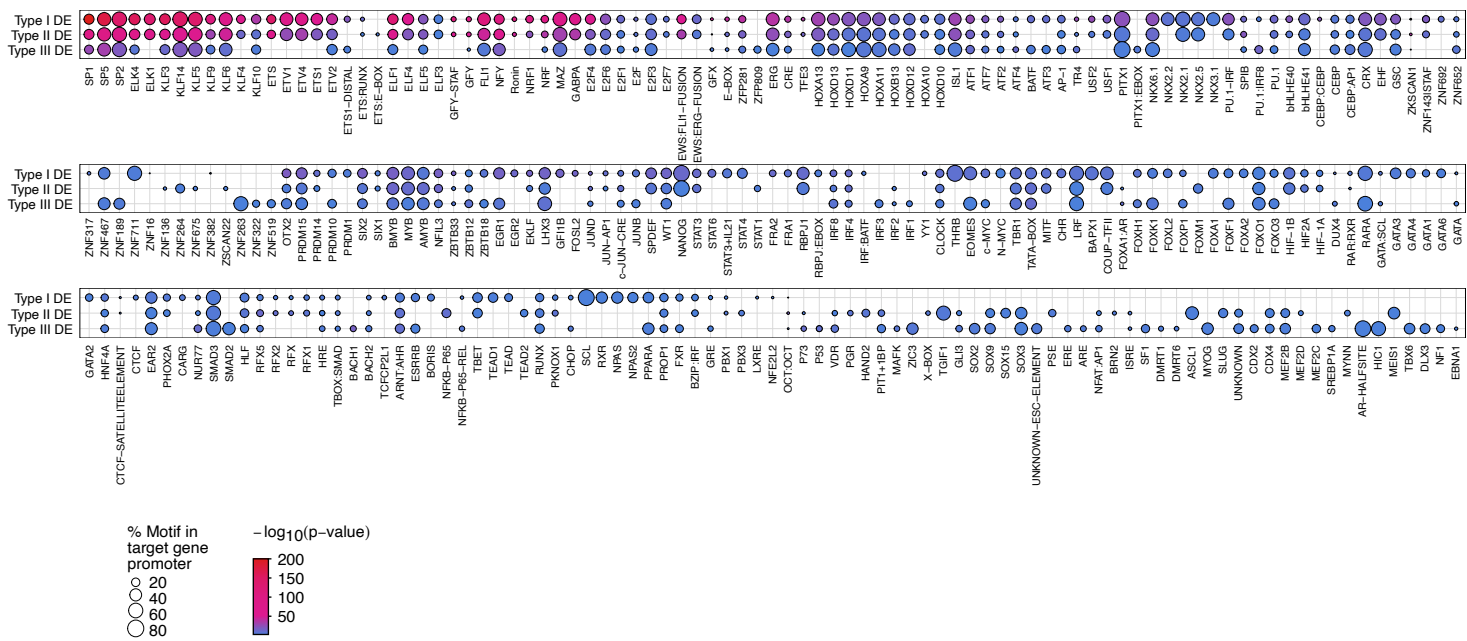

B

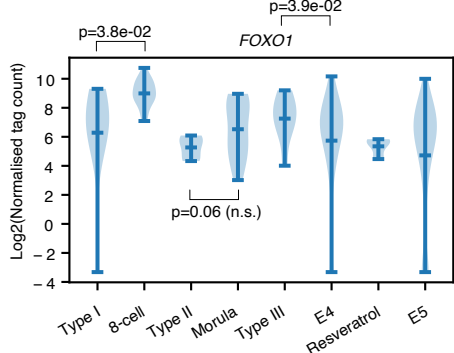

S10 Fig

Supplement: S10 Fig — Transcriptional regulation of embryonic arrest. (A) All significantly enriched motifs in the Types I–III arrested embryos in the promoters of DE genes. Motif discovery was performed using HOMER with default settings [98], against the promoters (defined here as −1,000 bp upstream) of the DE genes in the indicated types of arrested-embryo. The size of the circle indicates the percent of gene promoters that had the motif, and the color indicates the p-value for enrichment. (B) Violin plot showing the expression of FOXO1 in the indicated embryonic cell types. Significance is from a 2-sided Welch’s t test. Underlying data can be found in: https://figshare.com/articles/dataset/Human_embryo_normalized_gene_expression_data/19775992. n.s. = not significant. DE, differentially expressed. (PDF) [file pbio.3001682.s015.pdf]
