# Supplementary material for: Metabolic and epigenetic dysfunctions underlie the arrest of in vitro fertilized human embryos in a senescent-like state
Source: PLoS Biol. 2022 Jun 30;20(6):e3001682. doi: 10.1371/journal.pbio.3001682 (PMC9246109; doi:10.1371/journal.pbio.3001682)
Supplement: Attachment — Submitted filename: Response to Reviewers.pdf [file pbio.3001682.s016.pdf]

Editors’ comments:

Please provide the data underlying the graphs of the following figures:

Fig. 1A, B; Fig. 2A-I: Fig. 3A-J; Fig. 4A, B, D, E; Fig. 5A, D-I; Fig. 6B, C; Fig. S1A-E; Fig. S2A-D; Fig. S3A-D; Fig. S4A-E; Fig. S5A-G; Fig. S6A-E; Fig. S7A-D; Fig. S8A-E; Fig. S9A, B and Fig. S10B

Response: We have added several new data tables:

Data S1 – Values for several bar charts and figures in the manuscript, including: Raw data for Fig 1A, 1B, 2H, 3B, 3C, 4A, 4B, 5D, 5E, 5H, 5I, 5J, and Supplementary Figures S5A, S7A, S7C, S9A.
Data S2 – Normalized gene expression matrix for all samples and genes/TEs used in the study.
Data S3 – The raw tag count matrix of all samples (excluding resveratrol) used in this study for CytoTRACE analysis.
Data S4 – Karyotype proportion results.
Data S5 – All differentially regulated MZT genes, including gene rankings for GSEA plots.
Data S6 – All differentially regulated genes/TEs identified in this study.
Data S7 – Gene ranks for GSEA plots for all comparisons in this study.

All of the above data now have underlying supported data. Fig 3I, 3J, S6E were deleted in revision. We have added details to each figure legend for where the underlying data can be found.

Reviewers' comments

Rev. 1:
In the manuscript of Yang et al., the authors performed RNA-seq of arrested IVF human embryos in order to identify potential causes of developmental arrest. By comparing their RNA-seq data with published scRNA-seq of early human embryos, the authors classified the arrested embryos to three types according to their expression signature. They further demonstrated that the treatment of resveratrol or nicotinamide riboside partially rescued the embryo arrest through metabolic pathways. Overall, this is an interesting study providing potential explanations for IVF embryo arrest, however, detailed mechanistic study and additional controls are needed to support the conclusions.

Response: We appreciate the authors positive comments on our manuscript. To address the reviewers concerns we have added new data to the manuscript, and have carefully refined our claims in light of the reviewers’ comments. We have not been able to fully address all concerns, mainly due to limitations in access to biological material, but hope that the reviewer finds our revisions acceptable.

1. In line 18, single-embryo RNA-seq was used to identify the expression profiles, but multiple cells are present in a single arrested embryo (Figure 1). The developmental stage of each cell in an arrested embryo and the causes of the developmental arrest may be all different among cells. So the single-embryo RNA-seq only provide average expression signals from all cells within one embryo. So it is not accurate to classify the types of embryo arrests based on an averaged signal of different cells.

Response: We appreciate that this is a limitation of the study. However, we believe it is a reasonable compromise. Despite being an average of cells in the embryo the overall developmental stages can still be identified. This is likely due to the surprising heterogeneity in gene expression signatures in human embryos. For example, even at the late blastocyst stage when the epiblast, primate endoderm and trophectoderm emerge it can be challenging to identify the cells in single cell data. Additionally, on a technical level single-cell RNA-seq on blastomeres remains challenging to perform in humans due to the relative scarcity of material and the relatively high failure rate of single-cell RNA-seq on arrested embryos as they tend to disintegrate. Single-cell RNA-seq itself is also not necessarily ideal as it introduces artifacts in the sequencing, particularly transcript dropout, that are not present in samples consisting of multiple cells. Finally, we have been successful in identifying relatively subtle signature changes from bulk RNA-seq samples which are a large mixture of cell types (Hutchins et al., 2017). RNA-seq is especially sensitive to small changes in transcript abundance which produces signals that can be identified using methods such as PCA. Consequently, we feel that it is a reasonable compromise to consider single embryos. Nonetheless, to highlight this this concern, we have added a new paragraph to the discussion which describes this limitation in our study.

2. Cell number is correlated with developmental stages during pre-implantation development. Are there any relationship between the number of cells in arrested embryo and the arrested stage? Type I arrested embryos expressed 4/8-cell marker genes (line 145-146), but the authors claimed that Type I arrested embryos were at 2/4-cell stage according to Figure 2A. Please explain the reason that Type I arrested embryos are not assigned to 4/8-cell stage.

Response: This is an intriguing point, that we did not make clear. All of the arrested embryos were morphologically 2/4 cell stage, and they remained morphologically as 2/4 cell embryos. However, they adopted gene expression profiles and expressed specific marker genes from later developmental stages. Hence, in the arrested embryos the cell number is not correlated with developmental stage. We have revised the manuscript in several places to make this point clearer.

The Type I arrested embryos were assigned to the 4-cell stage based on the assessment of CytoTRACE (**Supplementary Figure 1C**), however considering the expression of many 8-cell marker genes, and indications that a faulty MZT had initiated (**Figure S3C, D**), we mainly compare the Type I arrested embryos with 4-cell and 8-cell stage embryos. We have revised the manuscript to make these points clear.

3. In Figure 2C, the authors claimed that ZGA genes were incompletely activated and maternal transcripts were not cleared. Given that each arrested embryo may contain cells of different developmental stages, the observed gene expression defects could be due to an averaged signal of a mixture of cells from different developmental stages instead of inefficient MZT in type I arrested embryos.

Response: We have added a section to the discussion that highlights this limitation in our study (see also Reviewer #1 point #1). The ZGA and MZT has been successfully detected and characterized from single embryo RNA-seq data (Sha et al., 2020).

4. In Figure 2H-I, the authors showed that TEs were activated but ZGA failed in type I arrested embryos. Are these activated TEs expressed in other developmental stages? What are the functions of abnormally activated TEs in type I arrested embryos and during development? Are the expression of TEs reversed by the addition of resveratrol or NR?

Response: These are intriguing questions, which we address separately:

Q1: The majority of up-regulated TEs (~80%) are specifically up-regulated in the Type I embryos. These TEs are not expressed or barely detected in any other embryonic stage. The remaining ~20% are expressed in at least one other normal embryonic stage, but are significantly up-regulated in the Type I arrested. We have added this data to the manuscript as **Figure S4F**. We believe this is a useful addition to the manuscript as it highlights the essentially uncontrolled release of TE expression. These TEs are not normally expressed in any developmental stage, which supports our argument that the cells have disrupted epigenetic regulation.

Q2: Only a limited number of TEs have been assigned any function in early embryogenesis, and most research has been performed using human embryonic stem cells, but those cells represent the late blastocyst. To our knowledge, no studies have shown a role for TEs in early embryo development and most work is extrapolated from mouse, which suggests LINE-1s may have a role (Percharde et al., 2018), as their RNA is also present at high levels in human blastomeres. None of the TEs expressed in the type I arrested embryos have an assigned function in embryogenesis, and as the answer above to Q1 indicates, the majority of up-regulated TEs in Type I arrested embryos are abnormal, and are not otherwise expressed in developing embryos. Consequently, we do not know if any of these expressed TEs have a function in early embryogenesis.

Q3: Yes, in the resveratrol-treated embryos the TEs were mainly down-regulated and were back to normal levels. Only a few TEs were differentially expressed between resveratrol and Type II and III, whilst versus Type I most TEs were down in the resveratrol-treated embryos (**Figure S7E, F**), and if we ranked TEs by their expression the overall levels of TE expression were close to normal embryos (**Figure S7F**). We have added this point to the manuscript.

Overall, we would like to thank the reviewer for these questions, which focus the TE part of the manuscript and help add data that supports our model for the deregulation of TEs in Type I embryos.

5. Figure 2H showed the activation of TEs in type III embryos, but Figure S4C and E showed otherwise. Could the authors explain? Is the activation of TEs in type I/III arrested embryos statistically significant in Figure 2H? The change of TEs may be unrelated to gene expression or ZGA. So it is necessary for the authors show expression for genes only (not genes and TEs together) between type I/II/III arrested embryos and embryos of different developmental stages.

Response:

Q1: This is an important and subtle point. Figure 2H and S4C and E are measuring different aspects of TE expression. TEs are difficult to measure as they exist as many distinct loci in the genome. Hence, to explore changes in their expression we interpret TE abundance from three perspectives: (1) all reads that map to any TE sequence fragment (**Figure 2H**), (2) TEs are divided into types (**Figure 2I, S4C**) and (3) in one case we measure the expression at all loci of a single TE type, LINE L1HS (**Figure S4E**). Consequently, it is possible to have differences in the total number of reads mapping to TEs that is not reflected in changes at specific TE types. For example, if high-expressed TE types are slightly up-regulated this would lead to increases in the total number of reads assigned to TEs, but may not lead to significant changes in TE types. Our data supports this idea, as only the Type I arrested embryos have substantial numbers of TE types specifically up-regulated (See Reviewer #1 Point 4 and **Figure S4F**).

To explore this further, we ranked all TEs by their expression in the Types I-III arrested embryos (Added as a new **Figure S7F**). Type I embryos show clear enrichment for TE expression levels, whilst the Type I and II arrested embryos show only elevated levels of TEs that are already relatively highly expressed. This suggests that Type I embryos have a global loss of TE regulation, whilst Type II, III and resveratrol-treated embryos have levels of TEs have only limited deregulation of a few types of TE (**Figure S4C, S7E**).

Q2: Yes, both Type I and III have significantly higher numbers of sequence reads assigned to TEs (we have modified **Figure 2H** to show this). However, whilst Type I show extensive TE deregulation of many hundreds of types of TE (**Figure 2I, S4F**), for Type III only a few TE types only a few TE types are significantly changed, and overall, there are few changes (**Figure S4C, S4F**). As described in Q1, the overall level is altered by a few high expressed TEs biasing the overall counts.

Q3: We have changed the volcano plots so that they now show genes and TEs separately (**Figure S4A, S5A, S5B, S8A, and S8D**).

6. Figure 3D only showed GSEA for senescence genes, not quiescence genes. The authors should not claim quiescence (line 293-294).

Response: We agree with the reviewer we are overstating our claims here, and have removed the claim of quiescence.

7. The authors showed that lower percentage of embryos located within G2/phase (Figure 3E). Again, even within the same embryo, each cell may be at different cell cycle stage. The authors cannot draw conclusions for cell cycle of arrested embryos based on current RNA-seq data. Other experimental approaches should be used to determine the cell cycle state of cells within an arrested embryo.

Response: Accurate measurement of the cell cycle in early embryos is challenging without transgenic reporters. We agree with the reviewer that calling an embryo as 'G1/S' or G2/M' implies a much higher level of precision than is possible from the data from the embryos. Our aim in this section is to demonstrate that the arrested embryos have reduced cell cycle activity. As this point is already exemplified by changes in the well-known cell cycle regulators *CDKN1A*, *CCNA2* (revised **Figure 3E**) and selected cell cycle gene expression (**Figure S6A, S6B**) we have removed the previous manuscripts' **Figure 3E**. We hope the reviewer agrees with this change.

8. The authors claimed that resveratrol rescued the arrested embryos, but why "the resveratrol-treated embryos still have deregulated levels of ribosomes, protein translation" and quiescence markers? This weakens the claim that cell cycle quiescence is the reason for developmental arrest.

Response: We agree this is somewhat unexpected. A possible explanation is that resveratrol is ultimately pushing the embryos into an unnatural state that forces development to recommence despite problems that persist in the embryos. We do not claim that the resveratrol-reactivated embryos are normal, indeed they continue to show disturbed gene expression profiles. We have added this point into the discussion to make our view clear.

9. "Resveratrol was pushing cells towards an increased glycolytic metabolism, whereas all the arrested cells had reduced glycolysis" (line 426-428) and Resveratrol activates Sirt1. But Sirt1 was reported to inhibit glycolysis, how to explain the discrepancy?

Response: We speculate that SIRT1 has different functions in embryonic cells, versus somatic cells. We see a change in fatty acid oxidation in response to resveratrol treatment, which agrees with previous studies showing a role for SIRT1 (Ding et al., 2017; Purushotham et al., 2009). However, we see the opposite effect of resveratrol on glycolysis. There is some evidence to support a different role of SIRT1 in embryonic cells. For example, Cha et al., showed that high SIRT1 expression is a feature of hPSCs which are primarily glycolytic (Cha et al., 2017), and inhibition of glycolysis leads to differentiation (Moussaieff et al., 2015). Cha et al speculated that SIRT1 may be exerting these effects not by directly regulating glycolytic enzymes, as occurs in somatic cells, but through an indirect process involving SIRT2 acetylation of glycolytic enzymes (Liu and Shyh-Chang, 2017). We speculate that SIRT1 is directly regulating fatty acid oxidation but is not regulating glycolysis in embryos.

Overall, we do not have enough information to determine the precise function of SIRT1 in the embryos, and we consequently reduce our claims in the manuscript about modulation of glycolysis through SIRT1. We have rewritten this section of the manuscript to more accurately reflect the ambiguity of the role of SIRT1 concerning overall metabolism.

10. The authors should upload all their single embryo RNA-seq data to public database and provide the link for reviewers to access. It is hard to judge the quality of the sequencing data and conclusions without accessible data.

Response: We apologie for not making this clear in the previous manuscript. The raw data for the RNA-seq is available at the GSA Human database https://ngdc.cncb.ac.cn/gsa/ with the accession number: HRA001406. The data is under controlled access to comply with Chinese government requirements on the sharing of human genetic data. The data will be made accessible to qualified researchers who make an application to the GSA Human system, as detailed in this document: https://ngdc.cncb.ac.cn/gsa-human/document/GSA-Human_Request_Guide_for_Users_us.pdf. Reviewers can inspect the submission using this temporary URL: https://ngdc.cncb.ac.cn/gsa-human/s/4MmpoTtE. The public GSA link will be released upon acceptance of the manuscript.

The quality of the RNA-seq data is high, and typically ~10,500 transcripts can be robustly detected (**Reviewer Figure 1**).

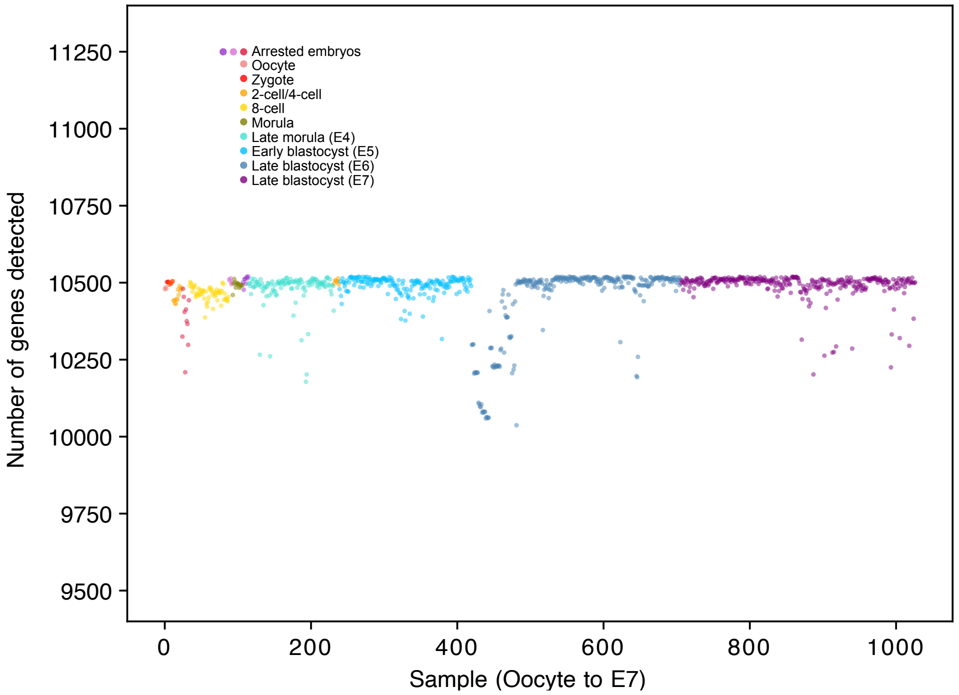

Figure 1 Number of genes detected in the indicated RNA-seq samples. A gene was considered detectable if its normalized tag count was greater than 30.

11. Important controls are missing. Morphology in control embryos should be added in Figure 1C and 4C. Immunostaining in control embryos should be added in 3I, 4F-G and 5B-C. Treating arrested embryos with solvent of Resveratrol and NR should be added as control.

Response: We have added morphology of untreated control embryos to **Figure 1C and 4C**. We have also added untreated control arrested embryos to **Figures 4F, 4G, 5B and 5C**. However, we do not have ethical approval to add immunostaining of untreated control presumed normal embryos at the same day as the arrested embryos for **Figure 3I, 4F, 4G, 5B, and 5C**. We appreciate that this is a potential weakness in our study, and we have highlighted this with the addition of a new paragraph in the discussion (Marked in blue in the track changes version of the manuscript). We have discussed this issue with the editor, and they agreed with this course of action, and we hope that the reviewer also finds this compromise acceptable.

12. Statistical analysis is lack, in all the figures, e.g. Figure 2D-F, 3A-F, 3G-J, 4E, 5A and all GSEA figures. NES values and FDR values are lack in all GSEA figures.

Response: We have added statistical tests for the appropriate comparisons for **Figures 2D-F, 3A-F, 3G-J, 4E, and 5A**, and their matching supplementary figures, where appropriate. We have added NES and FDR values to all GSEA figures.

Rev. 2:
In this manuscript, the authors investigated the molecular mechanism responsible for developmental arrest at different developmental stages during pre-implantation development of human embryos. They collected human embryos arrested at different stages and then performed single-cell RNA sequencing and bioinformatic analysis to further dissect the molecular events. By analyzing the RNA-seq data, they divided the arrested embryos into three types. The Type I embryos were arrested possible because of failure in zygotic genome activation. While the Type II and III embryos were arrested because of cell cycle progression

and metabolic problem. Finally, they found that the supplementation of resveratrol or nicotinamide riboside (NR) in the culture medium can partially rescue the arrested phenotype observed. Overall, the finding is interesting and should be publishable after revision.

Response: We thank the reviewer for their positive assessment of our manuscript.

1. They observed that ZGA is defective in Type I arrested embryos, which is predictable since these embryos were arrested at 2-5 cell stage and major ZGA occurred at 8-cell stage in human. Since the authors already found that ZGA genes were not activated and maternal genes expression remained very high, they can further discuss the possible reason causing this early stage arrest in human embryos.

Response: We thank the reviewer for drawing our attention to this point which was not adequately developed in the first submission. We have added new text (marked in blue) in which we speculate that the Type I arrested embryos have epigenetic regulation problems.

2. Both Type II and III arrested embryos exhibited cell cycle and metabolic defects, and it will be difficult for the reviewer to understand why the authors divided these arrested embryos into two types because they further presented their results together and no obvious difference can be observed anyway.

Response: We agree with the reviewer that it is unclear what the differences are between the Type II and III arrested embryos. We separate Type II and III based on **Figure 2B**, and because they show a relatively large number of differentially regulated genes between the two types (**Reviewer Figure 2**). Merging the analysis of Type II and III embryos leads to complexity as they adopt different developmental stages (**Figure 2A**). Consequently, although the Type II and III arrested embryos are similar in many ways, it is better to separate their analysis.

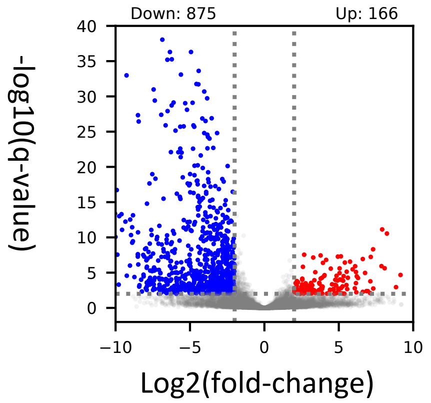

Figure 2 Volcano plot showing the number of differentially expressed genes between Type II and Type III arrested embryos. This plot only shows the differentially expressed genes. The x-axis is the log2 fold-change, and the y-axis is the -log10(q-value) as reported by DESeq2 with Bonferroni-Hochberg multiple testing correction. Significantly up and down-regulated genes are labelled in red and blue, respectively, and the number of genes that are up or downregulated is labelled.

References

Cha, Y., Han, M.J., Cha, H.J., Zoldan, J., Burkart, A., Jung, J.H., Jang, Y., Kim, C.H., Jeong, H.C., Kim, B.G., *et al.* (2017). Metabolic control of primed human pluripotent stem cell fate and function by the miR-200c-SIRT2 axis. Nat Cell Biol *19*, 445-456.

Ding, R.B., Bao, J., and Deng, C.X. (2017). Emerging roles of SIRT1 in fatty liver diseases. Int J Biol Sci *13*, 852-867.

Hutchins, A.P., Yang, Z., Li, Y., He, F., Fu, X., Wang, X., Li, D., Liu, K., He, J., Wang, Y.*, et al.* (2017). Models of global gene expression define major domains of cell type and tissue identity. Nucleic Acids Res *45*, 2354-2367.

Liu, T.M., and Shyh-Chang, N. (2017). SIRT2 and glycolytic enzyme acetylation in pluripotent stem cells. Nat Cell Biol *19*, 412-414.

Moussaieff, A., Rouleau, M., Kitsberg, D., Cohen, M., Levy, G., Barasch, D., Nemirovski, A., Shen-Orr, S., Laevsky, I., Amit, M.*, et al.* (2015). Glycolysis-mediated changes in acetyl-CoA and histone acetylation control the early differentiation of embryonic stem cells. Cell Metab *21*, 392-402.

Percharde, M., Lin, C.J., Yin, Y., Guan, J., Peixoto, G.A., Bulut-Karslioglu, A., Biechele, S., Huang, B., Shen, X., and Ramalho-Santos, M. (2018). A LINE1-Nucleolin Partnership Regulates Early Development and ESC Identity. Cell *174*, 391-405 e319.

Purushotham, A., Schug, T.T., Xu, Q., Surapureddi, S., Guo, X., and Li, X. (2009). Hepatocyte-specific deletion of SIRT1 alters fatty acid metabolism and results in hepatic steatosis and inflammation. Cell Metab *9*, 327-338.

Sha, Q.Q., Zheng, W., Wu, Y.W., Li, S., Guo, L., Zhang, S., Lin, G., Ou, X.H., and Fan, H.Y. (2020). Dynamics and clinical relevance of maternal mRNA clearance during the oocyte-to-embryo transition in humans. Nat Commun *11*, 4917.
